# The Problem (and the Value) of Radical Moral Disagreement

**DOI:** 10.12688/wellcomeopenres.24721.1

**Published:** 2025-09-09

**Authors:** David Lyreskog, Dominic Wilkinson, Caesar A. Atuire, Milly Farrell, Errol Francis, Mark Harrison, Patricia Kingori, Julian Savulescu, Ilina Singh, Michael Parker

**Affiliations:** 1 NEUROSEC, Department of of Psychiatry, University of Oxford, Oxford, England, UK; 2 Uehiro Oxford Institute, University of Oxford, Oxford, England, UK; 3 John Radcliffe Hospital, Oxford, England, UK; 4 University of Oxford Nuffield Department of Medicine, Oxford, England, UK; 5 University of Oxford Ethox Centre, Oxford, England, UK; 6 Culture&, London, UK; 7 Pandemic Sciences Institute, University of Oxford, Oxford, England, UK; 8 Faculty of History, University of Oxford, Oxford, England, UK

**Keywords:** Radical Moral Disagreement, Social Cohesion, Democratic Discourse, Polarization, Value Conflict, Ethical Pluralism, Moral Epistemology

## Abstract

**Background:**

Contemporary societies increasingly face deep, persistent moral conflicts that resist resolution through conventional dialogue and democratic processes. While the conceptual features of moral disagreement have been extensively analysed in philosophy, there has been inadequate attention to the full complexity of moral disagreements as social and cultural phenomena. This paper introduces and analyses the concept of "radical moral disagreement" (RMD) as a phenomenon requiring urgent scholarly attention.

**Methods:**

We conducted a conceptual analysis drawing on examples from contemporary moral conflicts including abortion debates, climate action, and pandemic responses. We identified and analysed seven defining characteristics of RMDs through theoretical examination and comparison with moral disagreements of other kinds. We developed a preliminary conceptual framework and outlined research questions spanning philosophy, history, sociology, psychology, and media studies.

**Results:**

We identified seven key characteristics that distinguish RMDs from ordinary moral disagreements: collision of incompatible values requiring resolution e.g. in policy decisions, intransigence even under ideal conditions for resolution, emotional intensity that precludes compromise, progressive polarisation of positions, censure that limits public discourse, widespread social disruption, and contested epistemologies regarding evidence and expertise. These characteristics are primarily scalar in nature, suggesting RMDs exist along a continuum rather than as categorical states. We demonstrated that RMDs can arise around a wide range of issues when they become entangled with identity, power, and risk within specific social contexts.

**Conclusions:**

RMDs represent complex moral and social phenomena that, while potentially destabilising social cohesion and democratic governance, may also catalyse moral progress when properly understood and navigated. The paper describes a theoretical foundation for studying RMDs and outlines relevant research questions requiring attention, spanning philosophy, history, sociology, psychology, and media studies. We propose that understanding RMDs is essential for developing effective approaches to coexistence in pluralistic yet polarised societies.

## Introduction

*The community of Scismoria is divided. It has a long history, and a rich cultural tradition. For centuries, Scismorians have enjoyed the practice of Treginda. Treginda is included in traditional stories and song. It has been taken to be a distinctive and central part of Scismorian culture. However, not all are comfortable with the practice. In recent years, those who oppose Treginda have become more vocal, and the practice deemed more controversial. A significant number of people within and outside Scismoria now regard it as deeply morally questionable. They raise a number of ethical objections and argue that it is offensive and distressing to even debate the continuation of this practice. Others claim that such views are scismoriphobic and deeply disrespectful of traditional Scismorian values and way of life. The national debate within Scismoria has become polarised and bitter, with protests occurring outside locations where Treginda is taking place or being discussed. Some of the national newspapers are strongly pro-Treginda, while others support the anti-Treginda cause. One national broadcaster attempts to represent both viewpoints whenever stories are raised but is frequently criticised for being biased. On social media, pro and anti Treginda astroturfing and trolling has become widespread. ^1^*

The world of Scismoria seems both alien and strangely familiar. The social and political worlds we live in seem more polarised than ever. There is a sense that deep and intractable value disagreements are increasing both in frequency and visibility, posing serious challenges for national and international peace and shared policy. ^2– 4^ Such disagreements inevitably have complicated origins. The impact of globalisation, migration, inter-generational value differences, social media and the wider digital world, increasing inequality – all of these and many others are likely to be impacting the acquisition, interpretation, and expression of viewpoints. ^5, 6^ While value disagreement and value pluralism are not new, contemporary disagreements are amplified by vast quantities of easily accessible information, disseminated at speed in ways that are profoundly asymmetrical. Social media create silos, distort arguments, and deepen divides. ^7^ These changes have produced profound polarisation in debates, exacerbated enduring differences and divisions, intensified value disagreements, and increased suspicious attitudes. ^8, 9^ Disagreements abound, even around subjective accounts of ostensibly 'shared' experience, while rising populist movements increasingly marginalise the voices of people from different backgrounds, or those whose life experiences or values differ from an identified majority. These disagreements nearly always have an important moral dimension. That is, they involve contested views about what should or should not be done or permitted, or about what is good, right or virtuous. This will often involve morally significant values that are linked to particular cultures or communities - but it may also involve views about the rightness or wrongness of certain practices, the balance of harms and freedoms, or the moral status of different entities. We appear to be living in an era of *radical moral disagreement (RMD)*.

Moral disagreement is not inherently bad. Indeed, deep moral disagreement may be seen not only as natural and unavoidable but also, in many cases, healthy and productive, giving rise to important new ideas and positive societal change, and what has often been understood as ‘moral progress’. ^10, 11^ Examples include the suffragettes, US civil rights movements, and anticolonial independence. Moral disagreement is likely to remain a pervasive feature of the worlds we live in and may in fact be a requirement for a healthy, flourishing society. ^12– 14^ But some forms of disagreement are impediments to rather than contributing to this.

## What are ‘radical’ moral disagreements?

The analysis of moral disagreement has been an enduring feature and core concern of both contemporary and historical moral philosophy. These debates have taken a wide variety of forms and have been concerned with a range of questions regarding the philosophically relevant features of moral disagreement, for example, the possibility of objective moral knowledge, arguments in favour of and against moral relativism and so on. Whilst often drawing upon and engaging with moral disagreements in the world outside philosophy, such as those about abortion or euthanasia, these debates have tended to pay inadequate attention to the full complexity of moral disagreements as social and cultural phenomena. More recently, primarily in epistemology but to some degree also in moral philosophy, there has been a growing interest in conceptualising and analysing the necessary and sufficient conditions for what, following Fogelin, have come to be known as ‘deep disagreements’. ^15^ Here too, however, whilst the debate about how best to conceptualise disagreements as ‘deep’ has been generative, and in some cases linked by authors to specific disagreements such as abortion, and vaccine confidence ^16^, the focus has been a concern with disputes about what constitutes good reasoning and less to the features of particular, stated moral disagreements. ^17^ These literatures have tended to pay little attention to the moral phenomenology of such disagreement, that is, to the variety and specificity of moral experience. This is a problematic omission, in that it misses something crucial to what drives identity formation and social disruption, and to what is at stake for those engaged in a world characterised by radical moral disagreement.

So, what is a radical moral disagreement? In what follows we develop a preliminary sketch of the key features of such disagreements, taking as our starting point the short list of candidate topics of radical moral disagreements in Box 1. We will return to discuss some of the limitations of this account further below, as well as explore its potential value as a starting point for a deeper critical engagement with these increasingly important social and political phenomena.

**Box 1. Examples of contemporary topics sometimes characterised by Radical Moral Disagreement**

Circumcision of infants or children before the age of consent

Return of artefacts from museum collections to original communities

Choices relating to termination of pregnancy or active ending of life

Vaccination mandates and lockdown in a pandemic

Gendered or religious dress codes – e.g. Burka, wearing of religious symbols in schools and public offices

Gender identity and sex change

Action to address climate change

Human rights, and violations of them

Regulation of online media platforms

Radical moral disagreements such as those in Box 1 typically display a number of common characteristics, including (but not necessarily all of) the following:

### Collision

Radical moral disagreement arises where incompatible views collide. Where there is pragmatic co-existence, or where those with conflicting views are separated in place and/or time, the problems of irreconcilable views do not typically arise in the same way. They frequently occur in situations where a common policy or action is needed or called for by legislators or public demand, and when no policy response can respect all opposing views adequately. For example, should Treginda be permitted, or should it be banned? In our example, no policy can satisfy both the anti- and pro-Treginda groups. Even the absence of a policy response is unsatisfactory, since it will result in either default proscription or permission. Compromises may be contested by both sides as involving unacceptable sacrifice of key (versions of) ethical values or principles. One party may feel that they are being obliged to uphold norms or values that they do not subscribe to or of which they are suspicious, while another party may feel that their views/position are so well grounded that they have a right or duty to ensure that everyone abides by them. Absence of a consensus will inevitably leave some members of the group feeling excluded or aggrieved.

### Intransigence

Radical moral disagreement endures in two important ways. The first of these is that such disagreements remain and are resistant even under what might be thought of as ideal conditions for their resolution. For example, the parties to the disagreement may all be well-intentioned and desire a resolution. The context of the exploration of such differences might be highly conducive to eventual agreement e.g., there may be no time-pressure. And yet, disagreement continues. A second way in which a radical moral disagreement might endure is that – perhaps counterintuitively – such disagreements are not always dissolved by making a decision or a policy, or even a law. If there were a decision – even democratically achieved - to ban Treginda, or to modify or limit it in some way, divergent moral views would likely remain. Of course, radical moral disagreements will not necessarily persist indefinitely. Over years, perhaps a generation or more, the disagreement over the original moral problem may fade. But such resolution, where it occurs, may nevertheless be ambiguous and open-ended, because the underlying moral issue may have transformed in the meantime.

### Intensity

Radical moral disagreements have a particular intensity, generating strong emotional responses both from those who engage directly in the disagreement, and from observers. Often, contrasting viewpoints are linked to certain forms of political, social or cultural identities in ways that have the potential to become emotionally and politically charged. This might be why those on either side (as seen in our Scismorian example) find the opposing viewpoint or any attempted compromise personally threatening, even existential. The intensity of RMDs also prevents compromise or even acceptance of harm reduction strategies. In the most dangerous cases this can result in violence.

### Polarisation

Radical moral disagreement is characterised by a tendency for views to gravitate towards binary and oppositional points of view. The more it is debated, the more RMD appears to become polarised. An example is the debate in the US around abortion, or the recent discussion on making puberty blockers available to children. ^18– 20^ Divisions and differences appear to deepen, and the possibility of agreement may seem to become ever less imaginable. The word ‘radical’ has another connotation here: those who disagree may become ‘radicalised’ and/or ‘essentialised’ in the way that they express their views. For these reasons, polarisation is closely linked to the formation of social, political and cultural identities. ^21^ It is both a cause and consequence of such identities and a person’s sense of self. Challenges to core values underpinning identities can therefore appear as existential. In 1936, H.G. Wells envisioned the "World Brain" as a new, free, authoritative, synthetic, permanent "World Encyclopaedia" that would enable global citizens to maximise the use of universal information resources and contribute to global peace. ^22^ Instead of fostering unity, serenity, and a shared understanding of reality, the internet seems to have had the exact opposite effect. Social media's greater voice has led to a decentralisation of information and the emergence of competing polarised narratives.

### Censure

As disagreement becomes more radical, it becomes more difficult to discuss or debate in the public sphere. ^23^ One or both sides may reject the expression of the opposing views on the grounds that such views are illegitimate, that they are manifestations of discrimination or prejudice and hurtful to the opposing group. There may be calls to limit, censor, ‘cancel’, ex-communicate or deny platforms to speakers with a particular perspective, and as seen in our hypothetical example, media organisations may struggle to facilitate discussion without risking offence – or actively participate in censorship. People taking particular positions may be socially stigmatised, and their views and positions intentionally misrepresented. But such responses of course do not remove the disagreement – they may simply push it out of the public space.

### Social disruption

Radical Moral Disagreements and their impacts tend not to be limited to discord and points of conflict between individuals or small groups. Rather, they play out on a larger scale within and/or between communities and entire societies, affecting wellbeing and causing disruption in the ways of life of parties directly or indirectly involved. Indeed, such disagreements lend themselves to overt politicisation. They have the capacity to energise and mobilise large swathes of the population. The prominence of RMDs in political struggles (and all political conflicts are to some extent moral conflicts) also tends towards greater use of violence and militarism to achieve political ends. ^24^ At a certain point, violence can come to seem like a moral necessity and not merely part of a political strategy. ^25^ One example is extreme environmental activism/eco-terrorism and animal rights activism, where individuals or groups feel compelled to act on behalf of those who cannot help themselves. ^26^

### Contested epistemologies

Radical moral disagreements often have a strong factual component. This will include disagreement not only about the factual claims themselves but also the question of who or what constitutes a reliable or trustworthy source of information. Consider, for instance, statements such as “the best way to address climate change is to prohibit the use of oil immediately”, or “the Covid-19 lockdowns were an overreaction”. These claims are factually formulated – they are contested descriptive statements about the world – but the reasons why people may agree or disagree with them are also value-laden in morally significant ways: perhaps they have opposing views on the priority of multi-species survival and wellbeing over time vs current human generations, and/or the importance of freedom vs public health, respectively. Furthermore, at the heart of moral disagreements there are frequently disputed factual claims. These may take many forms such as claims like ‘Treginda is harmful to individuals and communities’, or that some particular profession or media outlet is a (un)reliable source of information. It is useful to identify these factual claims, as they may point to places where further research or data may help to resolve questions or reach common understanding. However, such an effort may not always be successful. Where the facts relate to the distant past (for example in relation to historical claims), or to the future (predictions of the impact of particular actions on current and future generations), it may be practically impossible to resolve factual disputes. More importantly, whether as cause or consequence of radical moral disagreement, there may be disputed epistemologies and ontologies. Often, one or both sides may be deeply distrustful of particular types of evidence or approaches to gathering evidence. For instance, a particular mental phenomenon can be interpreted as evidence for a chemical imbalance or anomaly in cognitive function by one group, but as a difference or variation of normal, or spiritual (im)purity, or blessing/curse by others. Depending on which ontology one accepts, one may be inclined to partially or completely reject the evidence provided in favour of the other ontological position on the grounds that such evidence is unreliable or could not possibly be correct. A lack of trust in the character or intentions of the evidence-giver potentially further undermines attempts to seek resolution or compromise, engendering suspicion and unwillingness to engage on a common ontological and/or epistemic platform.

## Limitations of a scalar account of radical moral disagreement

In the above, we have outlined some of the recurring features that seem to characterise what we are calling radical moral disagreements (RMDs): collision, intransigence, intensity, polarisation, censure, social disruption, and contested epistemology. These features distinguish RMDs from more ordinary forms of moral disagreement. A key observation about these features is that most of them – perhaps with the partial exception of ‘collision’ – are scalar in nature. Disagreements may be more or less intense, more or less polarised, more or less disruptive, and more or less epistemically contested. Moreover, these features are not static. They are liable to change over time, sometimes intensifying and sometimes diminishing. A disagreement that begins as relatively contained may, under certain conditions, become radicalised. Conversely, even disagreements that initially seem intractable may, with time and contextual shifts, become more amenable to coexistence or even partial consensus.

This scalar conception suggests that radical moral disagreement may not be best understood as a categorical or binary state, but rather as an endpoint—or, more plausibly, a region—along a continuum. At one end of the spectrum, we find moral disagreements that are difficult, even deeply felt, but which still permit some degree of compromise, respectful disagreement, or pragmatic coexistence. At the other end are disagreements that exhibit many or all of the features listed above at a high degree and for which peaceful coexistence appears nearly impossible. Between these poles lies a diverse array of cases whose radicality is not immediately obvious and may be contested. This prompts an important question: is the difference between a radical moral disagreement and a ‘merely’ deep or persistent moral disagreement purely one of degree, or is it also one of kind? Is there, or ought there to be, a conceptual threshold for radicality?

It is tempting to introduce such a threshold. After all, some disagreements clearly seem to “go further” than others—they fracture communities, produce violence, or shape identity in ways that are deeply disruptive. But here, we encounter a conceptual and practical difficulty. To see this, consider the fictional case of Scismoria. On the face of it, the national dispute over the practice of Treginda appears to meet the defining criteria of RMDs. The disagreement is emotionally charged, highly polarised, resistant to resolution, and embedded in competing cultural identities and epistemologies. One might therefore be tempted to conclude that the disagreement is radical not merely in its social effects but in the moral seriousness of the issue itself. Perhaps, one might assume, Treginda involves some practice akin to abortion, euthanasia, or ritual scarification—issues widely recognised as morally weighty and culturally divisive.

But what if, on further investigation, it turned out that Treginda was more akin to the egg-breaking dispute satirised by Jonathan Swift in *Gulliver’s Travels*? ^27^ In Swift’s story, two neighbouring societies are embroiled in protracted and violent conflict over the question of whether eggs should be cracked at the big end or the small end. ^28^ Superficially absurd, the Lilliputian conflict parodies real-world disagreements that appear trivial to outsiders but are experienced as existential by those within. And crucially, Swift’s example prompts us to ask: must a disagreement concern an *objectively* morally serious issue in order to qualify as an RMD, or is the *experience* of seriousness by the disagreeing parties sufficient?

One plausible response is to say that even if the issue at hand is not, by some external metric, objectively important, the disagreement can still be radical if it is experienced and lived as such. On this view, radical moral disagreements are not defined solely by their content but also by their *function* and *phenomenology*. That is, they become radical through the way they are situated within social life: through their entanglement with identity, power, vulnerability, and risk. These divisions often take on moral valence, particularly in environments of perceived threat or competition.

This would suggest that radicality is not an intrinsic property of certain topics or practices, but rather an emergent property of specific social, cultural, and epistemic contexts. Abortion may be an RMD in some settings but not in others; the same holds for public health mandates, religious dress, or climate action. Indeed, even egg-breaking could become a radical disagreement in a context where it comes to symbolise broader struggles over tradition, autonomy, or collective identity. This has been extensively researched in Minimal Group Paradigm studies. ^29, 30^ Rather than being anomalies or mistakes, such cases illustrate that the threshold for moral seriousness – and for radicality – is often contested and dynamic.

Acknowledging the fuzziness of the concept of radical moral disagreement is perhaps a limitation, yet is also a diagnostic strength. It helps explain why some disagreements flare into crises while others remain dormant. It also cautions against overly simplistic attempts to categorise disputes as either “serious” or “trivial,” or simply “not.” More fundamentally, it reframes the task: instead of trying to identify which issues *are* RMDs, we might instead ask: *when*, *for whom*, and *under what conditions* do moral disagreements become radicalised? This shift opens the door to more empirically grounded and ethically attentive analyses of the dynamics of disagreement in pluralistic societies.

## Radical moral disagreements: key questions

In this brief, preliminary, and contestable conceptual analysis of the features of radical moral disagreements we have illustrated both the importance of complementing theoretical and conceptual work with empirical investigation, and the value of cross-disciplinary approaches. Our analysis also highlights the need for conceptual clarity to be interwoven with a sophisticated empirical analysis of roles of power and context in the emergence – or marginalisation – of radical moral disagreements. Making progress here calls for approaches that are theoretically, conceptually, and empirically engaged. Our analysis highlights that radical moral disagreements need to be understood as complex social and conceptual phenomena, and that is vitally important that we develop a nuanced understanding of them. It is intended as a starting point, a catalyst for discussion and, of course, disagreement. Drawing inspiration from the above discussion, in what follows we outline some of the ways in which the concept of ‘radical moral disagreement’ might offer a productive opportunity for critical reflection and debate about these increasingly common, troubling, and protean features of contemporary social and political life.

### Conceptual and philosophical work

There is scope for a great deal more philosophical and conceptual analysis here. For example, what defines Treginda? Are there different distinct forms of this practice? What good/values are those who in favour of Treginda trying to promote and is the practice the only way to achieve those ends? How should these be relevantly distinguished? Indeed, there will inevitably need to be discussion about what constitutes a serious moral issue, and what constitutes significant harm. In addition to the conceptual work required to get clearer about specific radical moral disagreements, this might include analysing how different philosophical approaches might offer resources for understanding and potentially bridging RMDs. This will need to include engagement with diverse philosophical traditions both within and beyond the Western canon if the development of an adequate philosophical understanding of moral disagreement and reconciliation is to be possible. Convening a diversity of approaches and understandings will be very important here. It will be particularly important to explore the relationship between radical moral disagreement and epistemic injustice, examining how power dynamics and contested epistemologies and epistemic injustices shape the recognition and legitimation of different moral viewpoints ^31^. A deeper and respectful engagement with the different moral traditions of the context where the RMD occurs will be required. It will be important to investigate whether RMDs represent genuine moral incommensurability or arise from deeper disagreements about the nature of moral truth and justification. That is, it will be important to make connections with debates in contemporary moral philosophy. At the heart of RMDs is frequently the issue of weighing different values, like liberty (and autonomy) versus well-being. Such weighing cannot be resolved by science but requires a philosophical approach such as application of theories, principles or arguments from consistency, the application of practical reason, or procedural approaches like reflective equilibrium ^32^.

### Historical and political analysis

RMDs do not materialise out of thin air but are the result of historical processes. It is therefore necessary to understand the factors which are conducive to their emergence and, in some cases, to their resolution or, at least, to effective mitigation. The circumstances in which such disagreements arise (e.g. governance structures, belief systems) vary widely but by analysing a range of case studies relating to a specific issue (e.g. the management of epidemics) it may be possible to identify common themes. Such an analysis may be helpful not only in highlighting factors behind the emergence of RMDs but also the political dynamics which tend to amplify such disagreements. Research in the field of political science suggests that ideological partisanship is inimical to deliberation and can thus entrench RMDs ^33^. A case in point is polarisation resulting from the recent Covid-19 pandemic. Crises of this kind tend to escalate RMDs because confrontation becomes more likely, producing interactional dynamics which are conducive to the crystallization of antagonistic social and political identities.

Understanding the process of when and why polarisation/RMDs occur and the mechanisms which have mitigated them are therefore vital. Having identified specific themes for historical investigation, we need to ask how the emergence and management of RMDs is affected by political leadership, social movements, their articulation in various media, among other things. Another crucial consideration is the policing and regulation of such disagreements. Where this is seen to be legitimate and balanced, disagreements and the conflicts arising from them can be diminished ^34^. If this is not the case, then disagreement and conflict can be aggravated. We might also ask how policing and deliberative/diplomatic traditions in different cultures have approached moral disagreement; from local peace-making practices to multilateral negotiations. Insights generated by such investigations could be applied to many contemporary challenges: not only those relating to epidemics but also other issues on which there are polarised views, e.g. artificial intelligence, biotechnology, antimicrobial resistance, where resolutions to competing moral frameworks and cultural traditions depend upon collective action and the finding of common ground.

### Experimental cultural spaces for radical moral disagreements

Due in part to the problems of biased and poorly reasoned thinking around RMDs there is an urgent need for systematic analyses applying existing models for addressing deep disagreement and polarization across different cultural and professional contexts. This might involve the study of traditional conflict resolution practices like the African Palaver Hut traditions, Indigenous talking circles, and Arab Sulha reconciliation processes. It will also include consideration of institutional/political approaches such as Truth and Reconciliation Commissions focusing on how they attempt to navigate profound moral differences while acknowledging historical injustices. Complementary to this, there is also value in analysing how different professions which involve engagement with seemingly intractable conflicts achieve their goals. This might include talking to professionals such as diplomats, international mediators, hostage negotiators, and marriage guidance counsellors among others. How do these practitioners create spaces for dialogue across deep divides, manage power imbalances, and build trust in high-stakes situations?

Building on insights from these existing models and experiences, work will need to be undertaken to design and evaluate new interventions specifically tailored to addressing RMDs and how different elements of successful conflict resolution practices might be adapted for moral disagreements that lack clear paths to resolution. It seems likely that many successful interventions will be interpersonal and in-person. However, it will also likely involve the development and evaluation of new digital tools and virtual spaces. Sophisticated evaluation methods will need to be developed to capture both immediate and long-term impacts of interventions, considering multiple stakeholder perspectives and different measures of success or failure across a range of settings. How digital platforms might be designed to support rather than undermine constructive engagement with moral differences? How can arts-based approaches complement other intervention methods and in particular look to develop and test non-verbal methods of engagement? How might approaches be scaled and adapted across contexts, from local community discussions to international negotiations? How might interventions need to be modified for different types of RMDs and different cultural settings? For example, museums, rather than being primarily being spaces for the storing and display of objects, could be one type of cultural space in which to develop new methodologies around how heritage practices can address political and social differences. The globalised dimensions of many issues with potential for radical moral disagreement mean that it is going to be essential to experiment with approaches with a global/international dimension.

### Sociological analysis

Any coherent account of radical moral disagreement will require investigation of the complex interplay of power structures and other structural phenomena that shape which moral disagreements become recognized as "radical" and which remain invisible or are suppressed. This will involve, for example, examining relations between “power” and “knowledge” to understand and analyse how media representation, and academic discourse privilege certain forms of moral disagreement while marginalising others. In addition, in the 21st century, we are witnessing important shifts in power and knowledge, which are no longer centrally controlled by a limited number of institutions. Rather, through large public platforms afforded by social media, the age of the expert is changing and ideas of expertise – as witnessed during the COVID-19 pandemic – are being openly contested. Accounts of radical moral disagreement will also need to analyse how institutional power, media representation, and academic discourse privilege certain forms of moral disagreement while marginalizing others. That is, considerations of epistemic and other injustices will be central.

It will also be important to study the phenomenology of living with radical moral disagreements across different social positions and power relations and to explore how marginalized communities experience and navigate moral conflicts that majority groups may not even recognise as controversial. This will involve examining how intersecting identities and power dynamics shape both the emotional burden of moral disagreement and the resources available for coping with it. It will also be important to document how communities resist the imposition of dominant moral frameworks while maintaining their own ethical traditions and ways of knowing, as well as paying attention to the ways in which such resistance can sometimes preserve forms of enduring inequity.

### Psychological research

There is already a wealth of psychological literature now on many of the features of RMDs, such as radicalization, and the formation of in-groups and out-groups, and it is important this is harnessed and further developed as part of the process of developing a deeper and more sophisticated understanding of radical moral disagreements. ^35^ Psychological research, especially social- and moral-psychological research, is important to understand how human biases, heuristics and psychological limitations may contribute to and perpetuate RMDs. But conversely, understanding these drivers better may also bring improved understanding and strategies for navigating RMDs in a constructive way.

### Media and communications research

As greater number of social media platforms introduce policies that seek to move away from strategies such as ‘de-platforming’, and at the same time remove their fact-checking operations, the landscape of what constitutes knowledge, “fringe views”, “fake news” and marginalised voices is rapidly evolving. Social science research on how these developments shape RMDs is important and will require a sophisticated digital component concerned with understanding social media and their interactions with other modes of communication. This will be crucial to our understanding of how different media and platforms shape the expression, amplification, and evolution of radical moral disagreements. How do media institutions frame moral conflicts and influence public understanding and engagement. How do social media algorithms and platform design affect moral discourse and polarisation? How do various stakeholders use media strategies to advance their moral positions or suppress opposing views? More positively, there will be need for research to consider how different communication technologies and formats might better support constructive engagement with moral differences, and develop and implement more effective misinformation management.

## Conclusion

Contemporary societies (like the fictional Scismoria) are increasingly characterised by morally significant disagreements that are difficult to resolve or address. In this paper, we have set out a preliminary account of some of the features of what we call “radical moral disagreements”. We have described these particular kinds of disagreements as having a set of common characteristics: collision, intransigence, intensity, polarization, censure, social disruption, contested epistemology, and concern with morally serious questions. Whilst the concept of RMD is vague and protean, it nevertheless picks out something important and deserving of greater attention and has the potential to act as a catalyst for new thinking and cross disciplinary collaborative research involving philosophy, psychology, sociology, politics, history, media studies and the arts, as well as novel experimental platforms for progressing dialogue in an age of polarisation.

## Data Availability

No data are associated with this article.

## Footnotes

^1^ It is perhaps worth noting at this point that readers of the case may intuitively assume Treginda to be analogous to specific controversial activities in the world. These might tend to be assumptions that Treginda is viewed by the reader as either an oppressive activity, or one that is liberatory. The case as presented is, however, deliberately neutral/agnostic in this regard.

^2^ Carpenter *et al.*, 2020.

^4^ Heltzel & Laurin, 2020.

^5^ O’Connor & Weatherall, 2019.

^6^ Pomerantsev, 2019.

^7^ O’Connor & Weatherall, 2019.

^8^ Rosenberg, 1992.

^9^ Policy Institute, King’s College London, 2020.

^10^ Lane, 2024.

^11^ Moody-Adams, 1999.

^12^ Parker, 2024.

^14^ There are, however, some traditions – both political and moral – that would disagree with this and consider the pursuit of harmony, and the minimisation of disagreement, as vitally important moral and/or political goals

^15^ Fogelin, 1985.

^16^ Ranalli & Lagewaard, 2022

^17^ Wee, 2025.

^18^ Latham, 2022

^20^ DiMaggio *et al.*, 1996.

^21^ Kohler, 2024

^22^ Wells, 2020.

^23^ This is complex. Whilst there will be an increase in ways in which contested topics and moral disagreements become difficult to discuss and speak about publicly in a reflective way, recent experience also suggests that there will at the same time be those e.g. on social media, in populist politics, and so on who will relish the intensification and polarisation of public debate,

^24^ Ginges, 2019

^25^ Hanh, 2023.

^26^ Hwang, 2021.

^27^ Swift, 1995.

^28^ In Swift’s book, the controversial edict requiring eggs to be broken only at the small end has its origins in a minor injury caused to the current king’s grandfather as a child when he tried to break an egg at the larger end. We are told that there have been several rebellions by members of the population who seek freedom to break their eggs at either end, whatever the risks.

^29^ Brown, 2020.

^30^ Diehl, 1990.

^31^ Fricker, 2007.

^32^ Savulescu *et al.*, 2021.

^33^ Strickler, 2018.

^34^ Stott *et al.*, 2020.

^35^ Haidt, 2001.
