# Supplementary material for: The Problem (and the Value) of Radical Moral Disagreement
Source: Wellcome Open Res. 2025 Sep 9;10:495. [Version 1] doi: 10.12688/wellcomeopenres.24721.1 (PMC12612615; doi:10.12688/wellcomeopenres.24721.1)
Supplement: Peer review history

# Reviewer report: Reviewer response for version 1

**DOI:** 10.21956/wellcomeopenres.27238.r142028

**Published:** 2026-01-16

**Authors:** Drew Johnson

**Affiliations:** 1 Independent searcher, Oslo, USA

**The Problem (and Value) of Radical Moral Disagreement -- Review**

 This paper highlights the need for interdisciplinary approaches and discussions on the topic of radical moral disagreement. The authors provide a useful framework for understanding the role or division of labor that different disciplines, including philosophy, history, sociology, psychology, and media studies can take in developing this concept further.

 Significantly, the paper also provides a corrective to philosophical approaches that tend to neglect the social and cultural context and embeddedness of radical moral disagreements. As the authors point out, this neglect is not simply an innocent abstraction away from merely contingent features of radical moral disagreements in order to get at their true essence--rather, by neglecting to account for the full complexity of radical moral disagreements in their social and cultural contexts, our understanding of them is limited and distorted. In these comments, I will focus on the authors discussion of the social and cultural embeddedness of radical moral disagreement, and some other critical points and suggestions.

**Disagreement and Relativism**

 While I find the emphasis on the social and cultural context of disagreement illuminating and important, I believe that a crucial topic underlying this discussion has been neglected: Namely, moral and epistemic relativism. A discussion of relativism is relevant for the paper because: 1) in the philosophical literature, the topic of deep disagreement is often considered in the context of arguments for and concerns about relativism (Carter 2014; Sankey 2010; Kolbel 2004; Hales 2014; Rorty 1979), yet where 2) among philosophers, relativism is almost universally rejected (Boghossian 2006; see Baghramian and Coliva 2020), whereas 3) among other disciplines that the authors engage with, such as sociology, relativism is accepted as a founding methodological assumption (Dilthey 1988; see Sera-Shrirar 2013).

 Indeed, the existence of deep disagreements, including radical moral disagreement, is taken by many as evidence in favor of relativism in morality or epistemology. The key thought here is that if a disagreement reflects different epistemic systems and is truly intractable under ideal conditions, this must be because there is no single, universal, objective rational framework that could settle the debate for once and for all. Rather, there must be multiple, incompatible epistemic systems yielding conflicting judgments, which cannot be neutrally evaluated and compared from a 'God's-eye' point of view. Proponents of relativism proclaim its virtues--its tendency to support humility and tolerance--as useful for addressing deep disagreements, which makes the view relevant for the authors to consider given their aims.

 However, relativism has faced fairly consistent resistance among philosophers (for over a thousand years--see Baghramian and Coliva 2020: Chapter 2) for purportedly being theoretically incoherent or self-refuting, and for leading to problematic results, such as endorsing tolerance for intolerable views. There are many arguments purporting to establish the incoherence of the view, but a major complaint is that relativism seems to at once capture the fact of disagreement--that incompatible epistemic or moral systems result in persistent conflict--while also making it impossible to explain disagreement, since each party seems to be saying only that their view is correct relative to their own respective epistemic systems, which is compatible with the correctness of the 'opposing' party's view according to their own system.

 In order to avoid relativistic implications, some philosophers writing on deep disagreement attempt to provide explanations for how disagreements might have the appearance of intractibility (or intransigence, in the authors terminology) without truly being intractable under ideal conditions (Pritchard 2009, 2011, 2018; Coliva and Palmira 2021). However, in idealizing away from actual circumstances in order to discuss how disagreement might be resolved in the epistemically ideal scenario, it is possible to miss the social and cultural situatedness that the authors (rightly, in my view) identify as central to the analysis of radical moral disagreement.

 Given their interest in fostering interdisciplinary conversations across fields with very different attitudes towards relativism, it may be wise for the authors not to endorse a specific stance on relativism, instead just raising some of these considerations for future work. However, a further concern is that some of the features of radical moral disagreement presented in the paper seem to already make relativist-leaning assumptions, and so the authors' model is not as theoretically neutral as it could be. In particular, the intransigence and the contested epistemologies features are stated in a way that favors relativism.

- Intransigence: According to the authors, "[RMDs] remain and are resistant even under what might be thought of as ideal conditions for their resolution". While these 'ideal conditions' are not defined, this statement is in tension with other philosophical discussions arguing that under ideal epistemic conditions, what appears to be a deep disagreement could in principle be rationally resolved. In turn, the impossibility of resolution under ideal conditions might be thought to entail epistemic relativism.
- Contested epistemologies: According to the authors, "[o]ften, one or both sides may be deeply distrustful of particular types evidence or approaches to gathering evidnce". This suggests a deep disagreement not only over what the facts are, but also what fundamental epistemic principles to use to discover the relevant facts (Lynch 2016). In turn, some have argued that an incommensurability in fundamental epistemic principles would entail relativism (Baghramian and Coliva 2020).

The authors might consider framing these features more neutrally, to avoid begging the question in favor of relativism.

**Questioning the Features of Radical Moral Disagreement**

 In my remaining comments, I raise some questions and critical considerations for the authors analysis of RMDs in terms of the seven features: 1) collision; 2) intransigence; 3) intensity; 4) polarization; 5) censure; 6) social disruption; and 7) contested epistemologies.

 While the authors emphasize the social and cultural contexts of RMDs, it may be useful to distinguish between features that are arguably inherent to the nature of the disagreement itself, versus features that are due to or contributed by the contexts in which they are embedded. In particular, it seems possible for conditions 1, 2, 3, and 7 to be described as inherent to the disagreement independently of the surrounding context (though the context may deepen or lessen how the feature manifests), whereas features 4, 5, and 6 seem to be primarily accounted for by the surrounding social context rather than initially driven by the disagreement.

 The authors claim that all the features they identify are scalar in nature, with the partial exception of 1. I did not find this claim fully convincing. Insofar as intransigence entails resistance to resolution even under ideal circumstances, that feature seems to be binary: either a disagreement is in principle resolvable under ideal epistemic conditions, or it is not. And insofar as contested epistemologies entails that a disagreement is due to a lack of shared fundamental epistemic principles, it also seems to admit of a binary characterization: either the interlocutors share an epistemic system and epistemic principles, or they do not. If some of these features are indeed less scalar than others, this could call into question the authors' claim that RMDs are not such because of some intrinsic quality, but rather because of their social context. See also my previous comment on how some of the features could be thought to be inherent. This result need not be a problem but could perhaps be integrated into the analysis. If some of the core features of RMDs are intrinsic while others are extrinsic, that could simultaneously capture the importance of social and cultural contexts and the phenomenology in determining whether a disagreement is an RMD, while also imposing some structural requirements a disagreement must meet in order to count. In short: while social, cultural, and phenomenological aspects may be necessary to an RMD, there may also be necessary intrinsic qualities. This could rule out Swift's egg-breaking disagreement, for instance, from being a genuine RMD, while also acknowledging the importance of interlocutors' experience and context in determining what is an RMD.

**Reference**

 Dilthey, W. (1988). Introduction to the Human Sciences. R.J. Betanoz, trans. London: Harvester Press.

 Sera-Shrirar, E. (2013). The Making of British Anthropology, 1813-1871. London: Pickering and Chatto.

Is the work clearly and accurately presented and does it cite the current literature?

Yes

If applicable, is the statistical analysis and its interpretation appropriate?

Not applicable

Are all the source data underlying the results available to ensure full reproducibility?

No source data required

Is the study design appropriate and is the work technically sound?

Yes

Are the conclusions drawn adequately supported by the results?

Yes

Are sufficient details of methods and analysis provided to allow replication by others?

Yes

Reviewer Expertise:

Social epistemology; hinge epistemology; epistemology of deep disagreement; epistemic virtue.

I confirm that I have read this submission and believe that I have an appropriate level of expertise to confirm that it is of an acceptable scientific standard.

## References

[1] : Relativism.2019; 10.4324/9780429343308 10.4324/9780429343308

[2] : Fear of Knowledge.2006; 10.1093/acprof:oso/9780199287185.001.0001 10.1093/acprof:oso/9780199287185.001.0001

[3] : Disagreement, Relativism and Doxastic Revision. *Erkenntnis* .2014;79(S1) : 10.1007/s10670-013-9450-7 155-172 10.1007/s10670-013-9450-7

[4] : Disagreement unhinged, constitutivism style. *Metaphilosophy* .2021;52(3-4) : 10.1111/meta.12490 402-415 10.1111/meta.12490

[5] : Motivations for Relativism as a Solution to Disagreements. *Philosophy* .2014;89(1) : 10.1017/S003181911300051X 63-82 10.1017/S003181911300051X

[6] : III-Faultless Disagreement. *Proceedings of the Aristotelian Society (Hardback)* .2004;104(1) : 10.1111/j.0066-7373.2004.00081.x 53-73 10.1111/j.0066-7373.2004.00081.x

[7] : After the Spade Turns: Disagreement, First Principles and Epistemic Contractarianism. *International Journal for the Study of Skepticism* .2016;6(2-3) : 10.1163/22105700-00603010 248-259 10.1163/22105700-00603010

[8] : Defusing epistemic relativism. *Synthese* .2009;166(2) : 10.1007/s11229-007-9278-2 397-412 10.1007/s11229-007-9278-2

[9] : Epistemic Relativism, Epistemic Incommensurability, and Wittgensteinian Epistemology.2011; 10.1002/9781444392494.ch14 266-285 10.1002/9781444392494.ch14

[10] : Wittgensteinian Hinge Epistemology and Deep Disagreement. *Topoi* .2021;40(5) : 10.1007/s11245-018-9612-y 1117-1125 10.1007/s11245-018-9612-y

[11] : Philosophy and the Mirror of Nature (1979).2020; 10.1007/978-3-658-16260-3_11-1 1-22 10.1007/978-3-658-16260-3_11-1

[12] : Witchcraft, Relativism and the Problem of the Criterion. *Erkenntnis* .2010;72(1) : 10.1007/s10670-009-9193-7 1-16 10.1007/s10670-009-9193-7

# Reviewer report: Reviewer response for version 1

**DOI:** 10.21956/wellcomeopenres.27238.r144032

**Published:** 2026-01-13

**Authors:** Rafael Ruiz

**Affiliations:** 1 London School of Economics and Political Science, London, UK

I found the article to be clear, careful, and largely uncontroversial in a positive sense. I didn't identify any obvious grounds for rejection. It succeeds in providing a useful conceptual framework for thinking about what the authors call “radical moral disagreement” (RMD), and the taxonomy of characteristics will likely be valuable for future interdisciplinary work on the topic.

 In particular, I found the discussion in *“Limitations of a scalar account of radical moral disagreement”* to be especially illuminating. The authors’ move to distinguish between the *function *and *phenomenology *of moral disagreement, which helps explain why some disputes come to be experienced as morally serious and socially destabilising even when, by more objective moral metrics (e.g. suffering, autonomy, harm), the underlying issue might appear comparatively minor. This is a genuine contribution and helps clarify why debates that may seem trivial to outsiders can nevertheless fracture communities, and viceversa, like how very urgent and important topics might be neglected.

 That said, this discussion also raises a point that may benefit from greater clarification earlier in the paper. The analysis seems to suggest that the radicality of moral disagreements is highly contingent on social, technological, and institutional factors (e.g. media environments, polarization dynamics). If so, it would be helpful to foreground this contingency more explicitly towards the beginning. This would also help distinguish the authors’ account from more epistemically focused treatments of deep disagreement, such as Wittgensteinian approaches (e.g. Lane 2024, which the authors cite).

 Relatedly, there is a slight tension between this socially contingent framing and later passages that emphasize more objective or structural moral factors, such as genuine moral incommensurability or conflicts between fundamental values (e.g. liberty versus wellbeing).

 For example, they say: *"It will be important to investigate whether RMDs represent genuine moral incommensurability or arise from deeper disagreements about the nature of moral truth and justification. That is, it will be important to make connections with debates in contemporary moral philosophy. At the heart of RMDs is frequently the issue of weighing different values, like liberty (and autonomy) versus well-being." *which seems to highlight *objective *rather than *phenomenological *factors. Clarifying whether RMDs are primarily radical due to contingent social conditions and phenomenology, or due to justificatory moral structure, (or whether the authors intend a hybrid view), would strengthen the coherence of the paper.

 The final sections of the article are good in setting out a wide range of promising questions for future research across philosophy and the social sciences. While the paper may not be highly innovative in the sense of introducing entirely new phenomena or arguments, it performs an important synthetic and agenda-setting function. As such, it works well as an entry point into the study of radical moral disagreement and as a platform for further interdisciplinary inquiry.

 Overall, I recommend acceptance, perhaps with minor revisions aimed at clarifying the relationship between social contingency, phenomenology, and moral structure in the authors’ account of radical moral disagreement. Just a few sentences in this regard would probably suffice.

Is the work clearly and accurately presented and does it cite the current literature?

Yes

If applicable, is the statistical analysis and its interpretation appropriate?

Not applicable

Are all the source data underlying the results available to ensure full reproducibility?

No source data required

Is the study design appropriate and is the work technically sound?

Yes

Are the conclusions drawn adequately supported by the results?

Yes

Are sufficient details of methods and analysis provided to allow replication by others?

Yes

Reviewer Expertise:

Moral Progress, Metaethics, Moral Epistemology, Normative Ethics

I confirm that I have read this submission and believe that I have an appropriate level of expertise to confirm that it is of an acceptable scientific standard.

## References

[1] : Deep disagreement across moral revolutions. *Synthese* .2024;204(2) : 10.1007/s11229-024-04710-z 10.1007/s11229-024-04710-z

# Reviewer report: Reviewer response for version 1

**DOI:** 10.21956/wellcomeopenres.27238.r134044

**Published:** 2025-10-22

**Authors:** J. Adam Carter

**Affiliations:** 1 University of Glasgow, Glasgow, Scotland, UK

This paper is a welcome addition to literature on deep disagreements. The authors correctly note that philosophical treatments of deep disagreement tend to focus on, e.g., epistemological, as well as also linguistic and semantic, aspects of such disagreements without theorising about them as a cultural phenomenon. And when paradigmatic contemporary cases of such disagreements (what they're calling RMDs) are viewed through this lens, a cluster of features that the authors identify stand out. That said there are are a few points that I think the authors would benefit from taking into account prior to publication.

 1) First, regarding the intransigence claim; it looks a bit strong to say that it's a feature of RMDs that they woudl be resistent to resolution even under ideal conditions for resolution. This looks tantamount to saying that these disputes are in principle intractable. It's not clear such a modally strong claim is needed to characterise what's interesting about RMDs;

 2) The section under contested epistemologies would benefit from further situating in relevant literature in epistemology where this kind of idea (vis-a-vis deep disagreements) is well studied; see, for instance, various papers in the recent handbook of Philosophy of Disagreement (Routledge, 2025) edited by Baghramian, Carter, and Cosker-Rowland;

 3) A more general point: it would be helpful to see a sharper articulation of what is *distinctively new* in the notion of RMD compared with existing treatments of “deep disagreement”. The authors are right to point out that RMDs can be viewed as a social phenomenon - but what additional explanatory power do we gain by working with the authors' framework, which we would lack if we limited ourselves to existing philospohical treatments of deep disagreement which we acknowledge (as Sunstein for example does - in discussing polarized disagreements) have social implications.

Is the work clearly and accurately presented and does it cite the current literature?

Yes

If applicable, is the statistical analysis and its interpretation appropriate?

Yes

Are all the source data underlying the results available to ensure full reproducibility?

Yes

Is the study design appropriate and is the work technically sound?

Yes

Are the conclusions drawn adequately supported by the results?

Yes

Are sufficient details of methods and analysis provided to allow replication by others?

Yes

Reviewer Expertise:

Analytic philosophy; epistemology

I confirm that I have read this submission and believe that I have an appropriate level of expertise to confirm that it is of an acceptable scientific standard.
